# My Country

**Published:** 1862-10

**Authors:** S. F. Smith


### MY COUNTRY.

BY REV. S. F. SMITH.

My Country! 'tis of thee,
Sweet land of Liberty,
Of thee, I sing:
Land where our fathers died,
Land of the pilgrims' pride,
From every mountain side,
Let freedom ring.

My native country! thee,
Land of the noble free,
Thy name I love;
I love thy rocks and rills,
Thy woods and templed hills;
My heart with rapture thrills,
Like that above.

Our fathers' God! to thee,
Author of Liberty!
To thee we sing;
Long may our land be bright
With Freedom's holy light:
Protect us by thy might,
Great God, our King!

God bless our native land,
Firm may she ever stand
Through storm and night;
When war's wild tempests rave,
Ruler of wind and wave,
Do thou our country save,
By thy great might.

OBEDIENCE TO THE AUTHORITIES.—The powers that be are ordained of God. Whosoever therefore resisteth the power, resisteth the ordinance of God: and they that resist shall receive to themselves damnation. Wherefore ye must needs be subject, not only for wrath, but for conscience sake.—Romans 13:1, etc. Submit yourselves to every ordinance of man for the Lord's sake: whether it be to the king, as supreme; or unto governors, as unto them that are sent by him for the punishment of evil doers, and for the praise of them that do well. For so is the will of God.—1 Peter 2 : 13, etc.

